# PRediction of acute coronary syndrome in acute ischemic StrokE (PRAISE) – protocol of a prospective, multicenter trial with central reading and predefined endpoints

**DOI:** 10.1186/s12883-020-01903-0

**Published:** 2020-08-27

**Authors:** Christian H. Nolte, Regina von Rennenberg, Simon Litmeier, Jan F. Scheitz, David M. Leistner, Stephan Blankenberg, Martin Dichgans, Hugo Katus, Gabor C. Petzold, Burkert Pieske, Vera Regitz-Zagrosek, Karl Wegscheider, Andreas M. Zeiher, Ulf Landmesser, Matthias Endres

**Affiliations:** 1 Klinik für Neurologie mit Experimentelle Neurologie, Charité-Universitätsmedizin Berlin, Hindenburgdamm 30, 12203 Berlin, Germany; 2 Center for Stroke Research, Berlin, Germany; 3 Berlin Institute of Health (BiH), Berlin, Germany; 4 Deutsches Zentrum für Herz-Kreislauf-Forschung e.V. (DZHK), 10785 Berlin, Germany; 5 Deutsches Zentrum für Neurodegenerative Erkrankungen (DZNE) – German Center for Neurodegenerative Diseases, 53127 Bonn, Germany; 6 Medizinische Klinik für Kardiologie, Campus Benjamin-Franklin, Charite-Universitätsmedizin, 12203 Berlin, Germany; 7 Klinik und Poliklinik für Kardiologe, Universitäres Herz- und Gefäßzentrum, Universitätsklinikum, 20246 Hamburg, Germany; 8 Institute for Stroke and Dementia Research (ISD), University Hospital, LMU Munich, 81377 Munich, Germany; 9 German Center for Neurodegenerative Diseases (DZNE, Munich) Partnersite, 81377 Munich, Germany; 10 Munich Cluster for Systems Neurology (SyNergy), 81377 Munich, Germany; 11 Klinik für Kardiologie, Angiologie, Pneumologie, Universitätsklinikum Heidelberg, 69120 Heidelberg, Germany; 12 Sektion für Vaskuläre Neurologie, Klinik und Poliklinik für Neurologie, Universitätsklinikum Bonn, 53105 Bonn, Germany; 13 Medizinische Klinik mit Schwerpunkt Kardiologie, Campus Virchow-Klinikum, Charite-Universitätsmedizin, 13353 Berlin, Germany; 14 Institut für Geschlechterforschung in der Medizin (Gender in Medicine, GiM), Charite-Universitätsmedizin, 10115 Berlin, Germany; 15 Institut für Medizinische Biometrie und Epidemiologie, Universitätsklinikum Hamburg-Eppendorf, 20251 Hamburg, Germany; 16 Klinik für Kardiologie, Angiologie, Nephrologie, Uniklinik Frankfurt, 60590 Frankfurt am Main, Germany; 17 Excellence Cluster NeuroCure, 10117 Berlin, Germany

**Keywords:** Acute ischemic stroke, Troponin elevation, Acute coronary syndrome, Heart-and-brain interaction, Stroke-heart-syndrome, Chronic coronary disease

## Abstract

**Background:**

Current guidelines recommend measurement of troponin in acute ischemic stroke (AIS) patients. In AIS patients, troponin elevation is associated with increased mortality and worse outcome. However, uncertainty remains regarding the underlying pathophysiology of troponin elevation after stroke, particularly regarding diagnostic and therapeutic consequences. Troponin elevation may be caused by coronary artery disease (CAD) and more precisely acute coronary syndrome (ACS). Both have a high prevalence in stroke patients and contribute to poor outcome. Therefore, better diagnostic algorithms are needed to identify those AIS patients likely to have ACS or other manifestations of CAD.

**Methods/design:**

The primary goal of the “PRediction of Acute coronary syndrome in acute Ischemic StrokE” (PRAISE) study is to develop a diagnostic algorithm for prediction of ACS in AIS patients. The primary hypothesis will test whether dynamic high-sensitivity troponin levels determined by repeat measurements (i.e., “rise or fall-pattern”) indicate presence of ACS when compared to stable (chronic) troponin elevation.

PRAISE is a prospective, multicenter, observational trial with central reading and predefined endpoints guided by a steering committee. Clinical symptoms, troponin levels as well as findings on electrocardiogram, echocardiogram, and coronary angiogram will be recorded and assessed by central academic core laboratories. Diagnosis of ACS will be made by an endpoint adjudication committee. Severe adverse events will be evaluated by a critical event committee. Safety will be judged by a data and safety monitoring board. Follow-up will be conducted at three and twelve months and will record new vascular events (i.e., stroke and myocardial infarction) as well as death, functional and cognitive status.

According to sample size calculation, 251 patients have to be included.

**Discussion:**

PRAISE will prospectively determine the frequency of ACS and characterize cardiac and coronary pathologies in a large, multicenter cohort of AIS patients with troponin elevation. The findings will elucidate the origin of troponin elevation, shed light on its impact on necessary diagnostic procedures and provide data on the safety and diagnostic yield of coronary angiography early after stroke. Thereby, PRAISE will help to refine algorithms and develop guidelines for the cardiac workup in AIS.

**Trial registration:**

NCT03609385 registered 1st August 2018.

## Background

Stroke and acute coronary syndrome (ACS) share similar vascular risk factors and may evolve as a complication of the respective other disease. One in five ischemic stroke patients will endure a severe cardiac event during the following weeks [1]. Acute coronary events such as myocardial infarction and sudden cardiac death account for about one third of these cardiac events and cardiac events are more likely to be fatal compared to recurrent stroke [2]. Identifying acute ischemic stroke (AIS) patients at high risk for coronary events may be a prerequisite for better prevention strategies in the future. However, few data exist of coronary screening strategies in AIS patients [3].

Troponins are the biomarkers of choice to test for myocardial injury [4]. Current guidelines by the American Heart Association/American Stroke Association (AHA/ASA) recommend troponin assessment in stroke patients [5]. This recommendation emphasizes that assessment of cardiovascular status is important in AIS patients. It refers to the robust observation, that elevation of cardiac troponin (cTn) indicates a higher risk for both mortality and poor outcome [6, 7]. Since cTn is a specific biomarker of cardiomyocyte injury, it is obvious to assume that cardiac diseases attribute to worse clinical outcome in stroke patients.

Both higher mortality and worse clinical outcome in AIS may at least in part be caused by concurrent myocardial ischemia due to obstructive coronary artery disease (CAD). In a small prospective study, coronary culprit lesions, defined as a lesion with irregular borders, filling defects, or the presence of intraluminal thrombus, and including high-grade stenosis leading to a reduction in Thrombolysis in Myocardial Infarction (TIMI) flow grade, were present in about 20–25% of AIS patients presenting cTn elevation above classical “rule-in” cut-offs for clinically suspected ACS [8]. On the other hand, nearly half of these AIS patients with relevant troponin elevation did not show any CAD at all. In those patients, stress induced neurocardiogenic dysregulation may contribute to cardiac injury following stroke (“stroke-heart syndrome”, for details see Scheitz JF et al. Lancet Neurology 2018) [9]. In support of this notion of stroke-induced cardiac injury, ischemic lesions in the right anterior dorsal insula representing the central autonomic network were significantly associated with dynamic troponin elevation as evidenced by voxel-based lesion symptom mapping [10].

Currently, relevant uncertainty remains regarding the underlying etiology and diagnostic consequences of cTn elevation in AIS. There is little evidence regarding diagnostic work-up and best medical care in this setting in AIS patients [11]. Present guidelines do not provide precise recommendations on the management of AIS patients with cTn elevation. Better diagnostic algorithms are urgently needed to identify those AIS patients likely to have ACS or other manifestations of CAD because therapies may be readily available.

Hence, the primary goal of the multicenter PRAISE study is to develop a diagnostic algorithm to better identify ACS as origin of cTn elevation in AIS patients. The primary hypothesis will test whether dynamic cTn levels (as compared to stable ones) indicate presence of ACS.

## Methods/design

### Study design, endpoints and safety assessment

The PRAISE Study is a multicenter, prospective, observational clinical trial with pre-defined endpoints. The predefined primary endpoint is diagnosis of ACS as stated by an independent endpoint adjudication committee (EAC) on individual patient basis. Predefined secondary endpoints are: Mortality at three and twelve months, new vascular events (stroke, myocardial infarction), degree of disability as defined by modified Rankin Scale (mRS) [12] at three and twelve months, cognitive status during hospital stay as defined by Montreal Cognitive Assessment (MoCA) [13], and cognitive status at three and twelve months as defined by Telephone Interview with Cognitive Status (TICS) [14].

Adjudication is based on clinical data and the reports from the central academic core laboratories that assess and report on diagnostic procedures. The core laboratories are blinded to clinical data. The EAC is blinded to cTn levels and changes over time to prevent a self-fulfilling prophecy. The EAC works independently of both the core laboratories and the steering committee. The adjudication process is subject to an EAC Charta, which is based on the current definitions of ACS. Current definitions of the ACS is represented by the European Society of Cardiology (ESC) guidelines [4].

Safety will be judged by a data and safety monitoring board (DSMB). The DSMB agreed on a DSMB Charta and will evaluate safety after inclusion of 100 patients, 200 patients and at study completion. Severe adverse events of special interest (SAESI) are recorded and adjudicated by a critical events committee (CEC). The CEC is independent of the study management. SAESIs incorporate death of any cause, stroke, myocardial infarction, bleeding according to the Bleeding Academic Research Consortium (BARC) classification type 3 to 5 [15], intracranial bleeding, and new coronary intervention during initial hospital stay.

In total, up to 30 academic and non-academic German centers will participate in PRAISE.

The websites of the study can be found at https://dzhk.de/en/research/clinical-research/clinical-studies/studie/detail/praisedzhk19idzneb001/ and at https://praise.dzhk.de/.

### Steering committee

According to good clinical practice, the PRAISE Study is overseen by a Steering Committee (SC) consisting of four Neurologists, six Cardiologists and one Statistician. The SC is updated regularly on study progress and gives expert advice to the study management to assure quality of the study. The SC considers the recommendations of the DSMB. In accordance with the recommendations of good clinical practice, the SC of PRAISE incorporates the Principal Investigators [16].

### Inclusion and exclusion criteria

Inclusion criteria are clinical diagnosis of ischemic stroke (all subtypes) based on neurological assessment and brain imaging (CT or MRI). Patients with the initial diagnosis of transient ischemic attack (TIA) may also be included, if initial focal neurological deficit has been verified by a board-certified neurologist. In addition, both risk of recurrence and likelihood of TIA have to be high, as defined by an ABCD^2^-Score ≥ 4 [17]. MRI is not obligatory to allow inclusion of patients with contraindications to MRI. Therefore, some patients with minor stroke maybe diagnosed as TIA.

Patients have to be at least 18 years of age and able to give written informed consent.

Time from symptom onset to hospital admission has to be less than 72 h. Elevation of high-sensitivity-cTn (hs-cTn) is defined according to the European Society of Cardiology guidelines for Non-ST-Elevation-ACS: i.e. highly-abnormal hs-cTn (i.e. > 52 ng/l if hs-cTn T, Elecsys-Assay, or > 52 ng/l, if hs-cTnI; Architect-Assay, or > 107 ng/l, if hs-cTn I. Dimension Vista Assay) or Δ-change at serial measurements depending on assay [4].

Patients with intracranial hemorrhage or cerebral sinus or cerebral venous thrombosis cannot be included in the PRAISE Study. Additional exclusion criteria are the following: renal insufficiency (eGFR < 30 ml/min/1.73 m^2^), uncontrolled thyroid dysfunction, known contraindications for coronary angiography (e.g. allergy to contrast agent), large ischemic lesion sizes > 100 ml on DWI-MRI or late CT or an ASPECT score of < 7 in case of early CT (within < 24 h) [18]. Further exclusion criteria are pregnancy or breast-feeding, limited life expectancy (< 1 year) or high premorbid degree of disability (mRS > 3). Previous myocardial infarction and other known manifestations of coronary artery disease are recorded and are no exclusion criteria because both may indicated high risk for ACS following AIS in particular. Coronary artery disease is defined as history of diagnosis (made by a physician) with one or more of the following criteria: Stenosis of coronary artery of > = 50% (diagnosed by coronary angiogram or other imaging method of coronary arteries), previous coronary bypass graft, previous percutaneous coronary intervention, and previous myocardial infarction of atherosclerotic origin. Application of i.v. thrombolysis and/or mechanical thrombectomy as well as mode of secondary prevention measures (dual antiplatelet therapy, oral anticoagulation) are recorded because aggressive treatment may increase the risk of troponin elevation [19].

### Procedures

All consecutive patients with the clinical diagnosis of stroke or TIA with ABCD2-Score ≥ 4 admitted within 72 h after symptom onset at the participating hospitals will be screened for pathological hs-cTn elevation and a dynamic change in hs-cTn levels. In case of study inclusion, the blood samples taken on admission and on follow-up (3–6 h thereafter) are saved, deep-frozen and biobanked. Biobanked samples will be (re-)analyzed in a central core laboratory using an identical hs-cTn assay. Analysis in the core laboratory will establish homogenously measured hs-cTn values and reduce heterogeneity of measurements between centers.

In addition, the PRAISE study takes part in the DZHK central biobanking program. This clinical research platform stores and contains biospecimens for subsequent analyses. This will offer the opportunity to investigate the role of cytokines (and other biomarkers of interest) in (nonischemic) myocardial injury in PRAISE.

Clinical scoring, coronary angiogram, echocardiogram and repeat ECG will be carried out according to Table 1. Coronary angiogram, echocardiogram and ECG will all be performed according to specified internal quality standards following a specific standard-operator-procedures (SOP) of the DZHK (Deutsches Zentrum für Herz-Kreislauf-Forschung; “German Centre for Cardiovascular Research”; DZHK; www.dzhk.de/en/). Diagnostic procedures do not have to be repeated if they have already been carried out in clinical routine before study inclusion.

**Table 1:** Frequency and scope of study visits

	Screening visit / Inclusion	Study procedures	Follow-up 1	Follow-up 2
	< 72 h after admission	< day 7 d	3 months	12 months
Inclusion/Exclusion criteria	X			
Informed consent	X			
**General examination**				
Medical history/comorbidities	X			
Physical examination	X			
Current Medication	X	X		
Neurological exam./NIHSS	X	X		
Degree of disability (mRS)	X	X	X	X
Psychological stress assessment (PSS)		X		
GRACE score	X			
Montreal Cognitive Assessment (MoCA)	X			
Severe Adverse Events of Special Interest (SAESI)	X	X	X	X
**Laboratory evaluation**				
Serial troponin	X			
Local blood chemistry	X			
**Diagnostic procedures**	**(assessment in central, blinded core laboratories)**			
Brain imaging (CT/MRI)	X			
12-lead surface ECG	X	X		
Echocardiography		X		
Coronary angiography		X		
**Endpoints**				
**Primary** Acute coronary syndrome (ACS)	Adjudication by Endpoint Adjudication Committee (EAC) based on clinical data and the reports of blinded core-labs			
**Secondary** **New vascular events and Safety**				
Stroke, TIA, MI, death, bleeding		X	X	X
		SAESI Independent assessment by critical events committee (CEC)		
**Cognitive Outcome**				
Montreal Cognitive Assessment (MoCA)		X		
Telephone Interview on Cognitive Status			X	X

*NIHSS* National Institutes of Health Stroke Scale, *mRS* Modified Rankin Scale, *ECG* Electrocardiography, *CT* Cerebral computed tomography, *MRI* Cerebral magnetic resonance imaging, *MI* Myocardial infarction, *TIA* Transient ischemic attack, *SAESI* Severe adverse event of special interest

A central study coronary core-lab - blinded for any clinical information - will analyze the following factors: Presence of CAD including number, location, lesion length, diameter of coronary lesions as well as a vessel-based assessment of the Syntax-Score, TIMI-Flow [19], AHA/ACC task force lesion type, Gensini Score [20], as well as presence of microvascular dysfunction including myocardial perfusion grade [21]. Furthermore, post-hoc functional characteristics will be analyzed including vessel- and lesion- based quantitative flow ratio (QFR) measurements [22].

Therapeutic coronary interventions as revascularization by PCI or CABG can be performed at the discretion of the treating physicians and will be recorded.

**Fig. 1:**
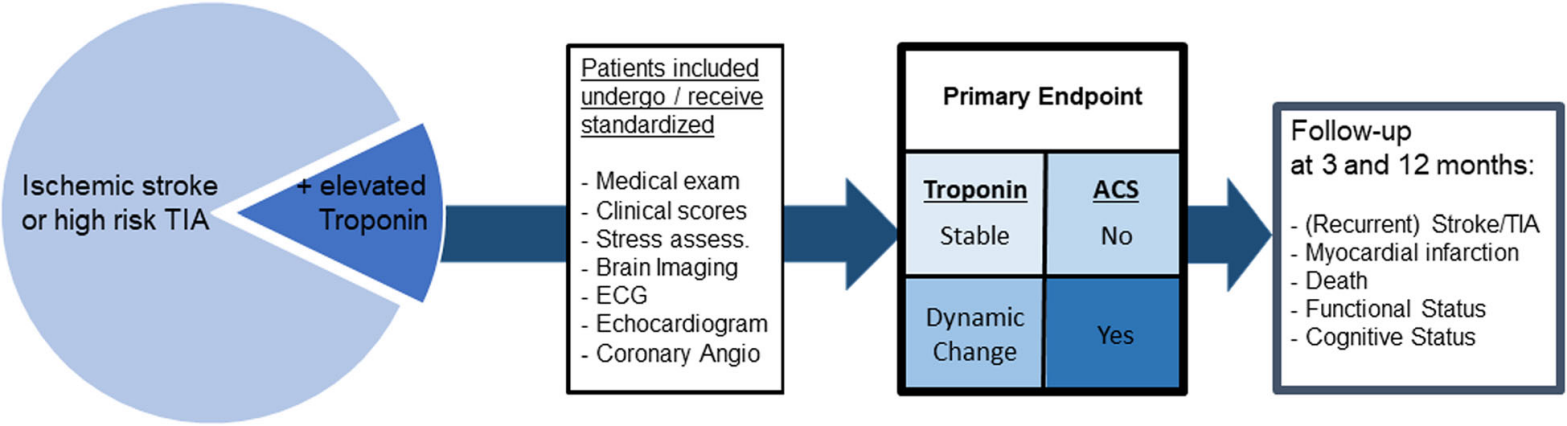
shows the study flow chart and visualizes the primary endpoint assessment and follow-up. ACS = acute coronary syndrome, ECG = electrocardiography, MI = myocardial infarction, TIA = transient ischemic attack

Transthoracic echocardiography is carried out according to the site imaging manual of the DZHK echo-core-lab Berlin (https://kardio-cvk.charite.de/forschung/berlin_cardiovascular_research_institute_bcri/services/echo_core_lab/) defining technical requirements, imaging and required views. Central raters are blinded to clinical information. Primary findings of interest include cardiac wall motion abnormalities as significant correlate of ACS and cardiac sources of embolism. The study flow chart is visualized in Fig. 1.

### Medical history, neurological and cognitive examination and clinical scoring

Patient characteristics will be recorded within an electronic case report file (eCRF) with SecuTrial® and contain age, sex, stroke risk factors including arterial hypertension, hypercholesterolemia, heart failure and atrial fibrillation, history of coronary artery disease. The eCRF can be assessed at (https://st03.mi.med.uni-goettingen.de/cgi-bin/WebObjects/productive-DataCapture.woa/wa/choose?language=de&customer=DZHK). Neurological examination will be carried out by certified investigators using the National Institutes of Health Stroke Scale (NIHSS) and the modified Rankin scale (mRS). Both instruments are widely used, validated and evaluated in German translation [12]. The NIHSS assesses stroke severity. The mRS assesses degree of disability. The Global Registry of Acute Coronary Events (GRACE) assesses prognosis and may be used to stratify for urgency of coronary angiogram. The MoCA will be used to assess cognitive status. The MoCA is available in German and has been validated in stroke patients [13]. The TICS allows assessment of cognitive status and has been validated in stroke patients, too [14, 23]. Telephone follow-up at 3 and 12 months will be performed centrally by experienced study personnel at the Charité Universitätsmedizin Berlin. The Perceived Stress Scale (PSS) will be applied to measure patients’ perception of stress [24]. A detailed list of study procedures can be seen in Table 1. In addition, an optional substudy will test whether women and men differ in presentation and outcome by employing a self-administered questionnaire.

### Statistics

The primary endpoint is presence/absence (Yes/No) of ACS in AIS patients in relation to presence/absence of dynamic troponin elevation.

The primary endpoint will be analyzed by chi-square test as both primary endpoint and primary test variable are dichotomous variables. Secondary endpoints will be analyzed using the chi-square test for comparisons of dichotomous variables of baseline characteristics and the outcome variables (ACS yes/no). The Wilcoxon signed rank test will be used for comparisons of continuous variables. Comparisons within the groups will be performed with the Fisher exact test and Mann-Whitney U test. We will calculate the area under the receiver-operating characteristics curves to evaluate the predictive accuracy of changes in troponin (and the other variables of interest) for the presence of ACS in stroke patients using the Harrell c statistic to determine the best cutoff to predict ACS. Thereby, a score to predict ACS can be created.

### Power calculation and sample size estimate

Assumptions for the sample size calculation are based on data from the prospective TRoponin Elevation in Acute Stroke (TRELAS) trial [8]. In TRELAS, 7/29 (24%) patients had coronary culprit lesion on coronary angiography and 11/29 patients (38%) had a dynamic troponin concentration pattern. Of those 11 patients with a dynamic troponin change, six had a coronary culprit lesion (6/11; 54%) whereas of those 18 patients who did not have a dynamic troponin change, only one had a coronary culprit lesion (1/18; 6%). The corresponding diagnostic odds ratio amounts to 20.4. As the overall sample size of TRELAS was small, we used the lower limit of the 80% confidence interval of the odds ratio which was rounded to 4 for the power calculation. The primary endpoint in PRAISE is ACS as assessed by the EAC (and not culprit lesion). However, all patients in TRELAS with culprit lesions also had ACS (and eventually underwent an intervention for treatment). Hence, assumptions for sample size calculation remain unchanged. Assuming that the percentage of patients with dynamic change observed in TRELAS will be approximated in the PRAISE study population, 203 patients will be required to reach significance (alpha = 0.05) with a power of 80%. However, if the percentage of patients with dynamic changes is between 0.38 and 0.5, smaller sample sizes are required to reach the same power. Thus, the sample size calculated on the basis of the TRELAS data will likely be sufficient for PRAISE. With 10% more patients to compensate for cases of misclassification and 10% for non-compliance or protocol violation (e.g. withdrawal of consent, violation of timing), 251 patients have to be included.

Compliance / Rate of loss to follow up: Adherence to medication is not an issue because we test a diagnostic approach. Loss to follow-up will be minimized by involving the central registration office (“*Einwohnermeldeamt*”). Rate of follow-up will by increased by standardized telephone-interview algorithm.

### Financing and external review

The PRAISE Study is financed by the German Centre for Cardiovascular Research (study code DZHK 19) and the German Center for Neurodegenerative Diseases study code (DZNE B001). The PRAISE study underwent a competitive, external review-process with the DZHK.

### Registration

The PRAISE study has been registered with clinicaltrials.gov: NCT03609385 on 1st August 2018 before inclusion of the first patient. The first patient in was included 6th August 2018.

## Discussion

The best approach to diagnose and treat acute coronary syndrome (ACS) in acute ischemic stroke (AIS) patients is unknown [11]. Presentation of signs and symptoms may differ in AIS patients due to impaired cognition, communication, sensation and perception. ACS may even be clinically silent in AIS patients. Guidelines recommend to measure troponin (cTn) in AIS patients, but diagnostic and therapeutic consequences remain vague [9, 11].

PRAISE will prospectively determine the frequency of ACS in a large, prospective, multicenter cohort of AIS patients with cTn elevation. By systematically acquiring and analyzing these data, PRAISE will test the hypothesis that dynamic troponin elevations (i.e. a “rise and fall pattern”) identifies concomitant ACS better than stable (chronic) troponin levels. Moreover, PRAISE will identify independent associations of other variables of interest with presence/absence of ACS. These variables include clinical scores, medical history, coronary pathologies, as well as signs of structural heart disease in AIS patients with elevated cTn. Thereby, PRAISE will provide a clinical algorithm for the diagnosis of ACS in AIS patients. These results will refine and improve current guidelines on diagnostic procedures and their consequences [5].

The thorough diagnostic work-up includes coronary angiogram as the diagnostic cornerstone and gold standard for establishing the diagnosis auf ACS [4]. Confirmation of diagnosis of ACS is achieved by coronary angiogram in the vast majority of patients presenting with chest pain. Coronary angiogram will also distinguish patients with type 1 from type 2 myocardial infarction, and the procedure will allow therapeutic interventions if a culprit lesion was identified [4]. In addition, coronary angiogram will delineate chronic coronary disease, which is associated with higher morbidity and mortality in AIS [25].

However, safety concerns have to be considered. Coronary angiography may require anticoagulation or prompt dual antiplatelet therapy due to stenting. Both early anticoagulation and dual antiplatelet therapy are associated with an increase in symptomatic intracranial hemorrhage in AIS patients [26, 27]. To acknowledge safety concerns, patients with large stroke volumes are excluded from the PRAISE study. Of note, data from the literature argue against a high risk of hemorrhagic transformation due to antithrombotic treatment [25]. Coronary angiography was performed in a large cohort of patients with ischemic stroke (without known troponin levels) and turned out to be safe even when performed in the early phase of acute stroke [25]. In any case, serious adverse events that might occur as complications of coronary angiography will be assessed by the critical events committee and monitored by the data and safety monitoring board and will be reported. Moreover, PRAISE will stratify patients according to timing of coronary angiography and investigate its potential effect.

Clinical evidence of chest pain or discomfort is not an obligatory inclusion criterion in PRAISE. ACS encompasses a broad and heterogeneous population ranging from patients presenting with chest pain, ST-elevation on ECG, and increase in cardiac biomarkers to those with atypical discomfort and non-specific ECG-changes [21]. Typical complaints may be absent in as many as 20% of patients [28]. A recent population-based cohort suggests that myocardial infarction may be “silent” (i.e. without pain or other complaints) in as many as 45% of patients [29]. Despite absence of overt clinical symptoms, these „silent “myocardial infarcts are also associated with higher mortality and morbidity [30]. In addition, “atypical” complaints are more often observed in the elderly, in women, in patients with diabetes, chronic renal disease, or dementia [31]. These characteristics are all common in stroke patients. Missing or atypical complaints may be even more common in AIS because stroke impairs cognition, communication, and sensation. Hence, in stroke patients clinical signs and symptoms may be severely disguised or absent. Finally, frequent comorbidities of AIS such as older age, diabetes, hyperlipidemia, hypertension, and previous manifestation of peripheral or carotid artery disease all increase the likelihood of ACS. Assessment of ACS in AIS patients remains a very demanding challenge and more data is needed to guide the clinician.

## Conclusion

The prospective, observational, multicenter PRAISE trial will assess the frequency of coronary and especially ACS-causing pathologies in AIS with troponin elevation. PRAISE will test the primary hypothesis that a dynamic change of cardiac troponin elevation allows identification of concomitant ACS in AIS. Furthermore, PRAISE will develop a diagnostic algorithm to diagnose ACS in AIS based on biomarkers, signs and symptoms, clinical scores, medical history, ECG, and echocardiography to identify those in need of a coronary diagnostics. Moreover, PRAISE will identify those patients with chronic CAD and high risk of future coronary events.

## Data Availability

The datasets used and/or analyzed during the current study will be available on reasonable written request.

## Abbreviations

ACS: Acute coronary syndrome
AIS: Acute ischemic stroke
CAD: Coronary artery disease
CEC: Critical event committee
CT: Computed tomography
cTn: Cardiac Troponin
DSMB: Data and safety monitoring board
DZHK: Deutsches Zentrum für Herz-Kreislauf-Forschung
DZNE: Deutsches Zentrum für neurodegenerative Erkrankungen
EAC: Endpoint adjudication committee
ECG: Electrocardiogram
eCRF: Electronic case report file
eGFR: Estimated glomerular filtration rate
hs-cTn: High-sensitivity cardiac Troponin
MI: Myocardial infarction
MoCA: Montreal Cognitive Assessment
MRI: Magnetic resonance imaging
mRS: Modified Rankin Scale
NIHSS: National Institutes of Health Stroke Scale
PSS: Perceived stress scale
QFR: Quantitative flow ratio
SAESI: Severe adverse event of special interest
SC: Steering committee
TIA: Transient ischemic attack
TICS: Telephone interview for cognitive status
TIMI: Thrombolysis in myocardial infarction
